# Pharmacokinetics of preoperative intraperitoneal 5-FU in patients with pancreatic ductal adenocarcinoma

**DOI:** 10.1007/s00280-021-04318-x

**Published:** 2021-06-16

**Authors:** Mikael Öman, Yvonne Wettergren, Elisabeth Odin, Sofia Westermark, Peter Naredi, Oskar Hemmingsson, Helena Taflin

**Affiliations:** 1 Department of Surgical and Perioperative Sciences, Surgery, Umeå University Faculty of Medicine, Umeå University, 90185 Umeå, Sweden; 2 Department of Surgery, Institute of Clinical Sciences, Sahlgrenska Academy at University of Gothenburg, Sahlgrenska University Hospital/Östra, 41345 Göteborg, Sweden; 3 Department of Surgery, Örnsköldsviks sjukhus, 89135 Örnsköldsvik, Sweden

**Keywords:** 5-Fluorouracil, Intraperitoneal chemotherapy, Pancreatic cancer, Pharmacokinetics, Gene expression

## Abstract

**Purpose:**

The aim was to investigate the pharmacokinetics of preoperatively administered intraperitoneal (IP) 5-FU in patients with resectable pancreatic ductal adenocarcinoma (PDAC) by analyzing levels of 5-FU and target metabolites in peritoneal fluid, plasma, liver, lymph nodes, pancreatic tumour, and pancreatic tissue. These results were correlated to expression of genes encoding enzymes of the 5-FU pathway and cell membrane transporters of 5-FU and FdUMP.

**Methods:**

Twenty-two patients with PDAC were treated with IP 5-FU before surgery. The postoperative treatment followed a routine clinical protocol. 5-FU and its metabolites were analyzed by LC–MS/MS. The expression of genes encoding enzymes and transporters in the 5-FU pathway was analyzed by qPCR.

**Results:**

After IP treatment, 5-FU could be detected in plasma, lymph nodes, liver, pancreatic tumour, and pancreatic tissue. The highest 5-FU concentration was found in the liver, also expressing high levels of the 5-FU transporter OAT2. 5-FU was converted to active FdUMP in all tissues and the highest concentration was measured in lymph nodes, liver and pancreatic tumour (18.5, 6.1 and 6.7 pmol/g, respectively). There was a correlation between the FdUMP and dUr levels in lymph nodes (*r* = 0.70, *p* = 0.0076). In tumours, there was an association between OAT2 expression and FdUMP concentration.

**Conclusion:**

The study shows uptake of IP 5-FU and drug metabolism to active FdUMP in pancreatic tumour, liver, and lymph nodes. Extended studies are warranted to evaluate the IP route for 5-FU administration in PDAC patients.

**Supplementary Information:**

The online version contains supplementary material available at 10.1007/s00280-021-04318-x.

## Introduction

The incidence of pancreatic ductal adenocarcinoma (PDAC) is increasing worldwide [1] and despite advancements in detection and management, the 5-year survival rate is around 9% [2].

The only cure for PDAC is radical surgery, performed as a pancreatoduodenectomy for tumours located in the head of pancreas, or distal pancreatectomy for tumours located in the body or tail. The disease is often detected in an advanced stage, leaving less than 25% of the patients suitable for radical surgery [3]. Of these only 25% will survive more than 5 years [4].

Despite radical surgery, 75% of the patients will within 2 years, develop a relapse mostly localized in the liver, in the bed of resection or in the peritoneum [5]. The high degree of local recurrence has been explained by the aggressive tumour biology with early lymph node metastases and microscopic resection margin involvement (R1) in up to 40% of the patients [6, 7].

Current guidelines recommend adjuvant chemotherapy after surgery for PDAC. A modified adjuvant FOLFIRINOX regimen (5-fluorouracil, leucovorin, irinotecan, oxaliplatin) results in improved overall survival compared to gemcitabine [8] at the expense of increased toxicity. Neoadjuvant FOLFIRINOX, which increases resectability of borderline resectable PDAC and improves survival in palliative patients [9], is currently investigated in a clinical trial for resectable PDAC [10]. The PREOPANC-2 trial compares neoadjuvant FOLFIRINOX with gemcitabine-based chemoradiotherapy and adjuvant gemcitabine, in patients with resectable PDAC [11]. Thus, combination therapies containing 5-fluorouracil (5-FU) are used in both neoadjuvant, adjuvant, and palliative treatment regimens for PDAC [12].

The frequent locoregional relapses after radical surgery for PDAC and the toxicity of modern combination chemotherapy regimens raise the need of novel treatment strategies [13, 14]. The stromal component in pancreatic cancer might mitigate intravenously administered drugs to reach the target cancer cells. Intraperitoneal (IP) administration has the potential of yielding a higher total drug exposure (Area Under Curve) for the intraperitoneal compartments than if the drugs are given IV [15]. In a porcine model, IP 5-FU resulted in high concentrations in peritoneum, compared to plasma [16]. In human cancer [17], and specifically in PDAC [18], IP 5-FU is safe in doses up to 1250 mg/m^2^.

**Fig. 1:**
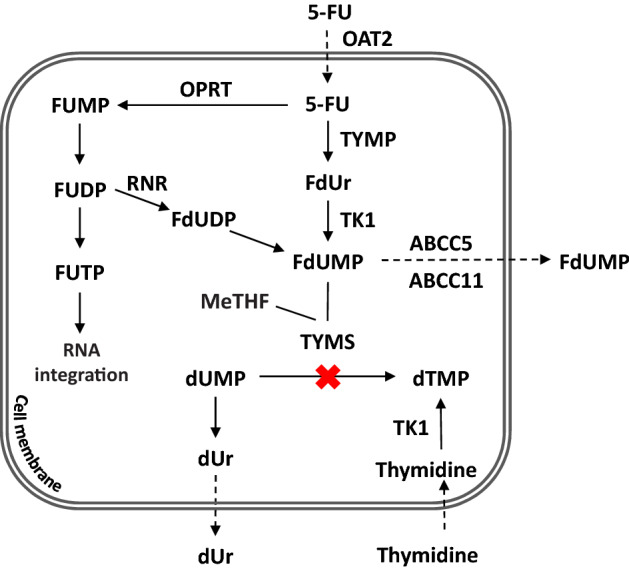
Simplified schematic of the intracellular conversion of 5-FU to FdUMP. 5-FU is converted to the active metabolite FdUMP by two main routes. In one route, the enzyme thymidine phosphorylase (TYMP) catalyzes the conversion of 5-FU to fluorodeoxyuridine (FdUr), which in the next step is phosphorylated to FdUMP by thymidine kinase (TK1). In the other route, the enzyme orotate phosphoribosyltransferase (OPRT) converts 5-FU to fluorouridine monophosphate (FUMP) which subsequently is phosphorylated to fluorouridine diphosphate (FUDP) by kinases. The enzyme ribonucleotide reductase (RNR) then converts FUDP to fluorodeoxyuridine diphosphate (FdUDP) which is dephosphorylated to FdUMP

Cellular uptake of 5-FU is mediated by the organic anion transporter 2 (OAT2) (Fig. 1) and high OAT2 expression correlates with good response to neoadjuvant 5-FU-based chemotherapy [19, 20]*.* After entering the cells, 5-FU is converted to the active metabolite fluorodeoxyuridine monophosphate (FdUMP) [21], which forms an inhibitory ternary complex with thymidylate synthase (TYMS) and the cofactor [6R]-5,10-methylenetetrahydrofolate (MeTHF). This results in inhibition of de novo dTMP synthesis and subsequent impairment of DNA synthesis and repair. TYMS inhibition causes a rise in the intracellular pool of the natural TYMS substrate deoxyuridine monophosphate (dUMP), leading to increased levels of deoxyuridine (dUr) in tissues and blood [22]. However, FdUMP can be transported out of cells by the drug efflux proteins ABCC5 and ABCC11 and upregulation of ABCC5 in pancreatic cell lines is associated with development of 5-FU drug resistance [23, 24]. Activation of the salvage pathway, in which thymidine is converted to dTMP through the action of TK1, may also contribute to 5-FU resistance [21]. In addition to inhibiting TYMS, 5-FU can act by incorporating fluorinated derivatives into the RNA and DNA of tumour cells. However, the association between this incorporation and the effect of 5-FU treatment appears to be weak compared to TYMS inhibition [25].

The aim of study was to investigate the pharmacokinetics of preoperatively administered IP 5-FU in patients with radiologically resectable PDAC, by analyzing levels of the drug and its metabolites in tissues and plasma, to demonstrate whether IP 5-FU results in drug uptake and metabolism to FdUMP in PDAC and adjacent tissues within the metastatic route. The results were correlated to the expression of genes encoding enzymes of the 5-FU pathway and membrane transporters of 5-FU and FdUMP.

## Patients and methods

Patients with radiologically resectable PDAC were included. Preoperatively, a laparoscopy was performed to exclude peritoneal metastasis and to implant a Port-a-cath (JCL Technic, Vallentuna, Sweden) subcutaneously over the right costal edge, with the catheter free floating in the abdominal cavity. The night before pancreatic surgery, 5-FU (Flurablastin^R^, Pharmacia Sverige AB, Stockholm) 1250 mg/m^2^ diluted in 2000 ml room tempered 0.9% sodium chloride (NaCl), was instilled by gravity in the Port-a-cath for 60–120 min. Folinic acid 100 mg/m^2^ (5-formyl-tetrahydrofolate, Teva Sweden AB, Helsingborg, Sweden) diluted in 1000 ml 0.9% NaCl, was administrated intravenously for 60 min by infusion pump, 30 min after the start of 5-FU instillation, to avoid intraperitoneal chemical reaction before cellular uptake.

To extract and sample IP fluid before laparotomy, a stiff suction catheter was inserted in abdominal cavity through a small subumbilical incision. To remove remaining fluid, 2000 ml body tempered 0.9% NaCl were installed through the suction catheter. The patient was rotated from one side to the other to optimize dispersal, and the wash fluid was extracted and sampled for analysis.

Immediately after laparotomy, lymph nodes from station 8A and/or 12A and a knife biopsy from liver segment 3 were sampled. After resection, a knife biopsy from the pancreatic tumour and tissue was sampled. If only an exploratory laparotomy was performed, no sample of pancreatic tumour and tissue was obtained.

The postoperative treatment followed a routine clinical protocol. Surgical complications within 30 days were characterized according to the Clavien–Dindo scale [26]. A Clavien–Dindo complication grade 1 is any deviation from a smooth postoperative recovery, and may require pharmacological treatment. Grade 2 includes total parental nutrition and blood transfusions. Grade 3 requires intervention in no or local (3A) or general (3B) anaesthia. Grade 4 is life-threatening requiring intensive care unit management due to single organ failure (4A) or multiorgan dysfunction (4B). Grade 5 is fatal.

Laboratory and symptomatic adverse events due to IP 5-FU were evaluated according to Common Terminology Criteria for Adverse Events (CTCAE version 5.0) [27]. A CTCAE grade 1 is mild, grade 2 moderate, grade 3 severe, grade 4 life-threatening or disabling and grade 5 fatal. Long-term follow-up consisted of clinical check-up 1, 3, and 6 months after surgery and then biannually.

Blood samples were drawn at start of laparotomy and immediately after pancreatic resection or, if exploratory laparotomy, when closing the abdomen. Venous blood samples were collected in EDTA vials, and plasma was isolated within 30 min by centrifugation at 2000xg for 10 min. The plasma was stored at − 80 °C until analysis. Tissue biopsies were instantly put on dry ice, then snap-frozen in liquid nitrogen and stored at − 80 °C until used.

### Preparation of plasma, IP fluid, and wash fluid

Proteins were precipitated by mixing 300 µl plasma with 1 ml 90% ice-cold methanol for 10 min at 8 °C. After mixing, 10 µl of the internal standard chlorodeoxyuridine (CldUr, 0.05 mM) were added. The mixture was vortexed and centrifuged at 21500× *g* for 10 min at 8 °C. The supernatant was analyzed with Liquid Chromatography with tandem Mass Spectrometry (LC–MS/MS). For calibration purposes, known amounts of 5-FU, dUr, and FdUr were spiked into a blank plasma sample, and handled as described. The IP and wash fluids were analyzed directly on the LC–MS/MS.

### Preparation of tissue samples

The tissue was weighed and placed in an Eppendorf vial, and 700 µl ice-cold 90% methanol were added. Homogenization was performed on a Retsch TissueLyser (Qiagene) using two disruption steps at 25 Hz for 2.5 min with freezing of the samples in between. Next, 10 µl of the internal standard CldUr (0.5 mM) were added, and the sample was mixed and centrifuged at 3000× *g* for 5 min at 8 °C. The supernatant was saved and the remaining tissue was mixed with an additional 700 µl 90% methanol followed by homogenization. After centrifugation, the two supernatants were pooled and evaporated into dryness. Thereafter, 200 µl of distilled water were added followed by centrifugation at 21500× *g* for 10 min at 8 °C. For calibration, known amounts of 5-FU, dUr, FdUr, FdUMP, and dTMP were spiked into a blank biopsy homogenate with internal standard, handled as described. Two separate calibration curves were constructed; one for 5-FU, dUr, and FdUr, and one for FdUMP and dTMP.

### LC–MS/MS analysis

LC–MS/MS analysis was performed on a Waters 2795 LC separation module coupled to a Micromass Quattro Triple-Quadrupole MS system with an electrospray ionization source. The source conditions were: temperature at 140 °C, desolvation gas temperature at 350 °C, cone voltage at 26 V, and capillary at 3 kV. All metabolites were tuned in electrospray negative mode. The MS/MS acquisition method was optimized for maximum response for the 5-FU metabolites (SI Table 1). The separation was performed using an Atlantis dC18 3 µm, 2.1 × 100 mm column, together with an Atlantis guard column dC18 3 µm, 2.1 × 10 mm. The mobile phase for 5-FU, dUr, and FdUr consisted of 5 mM acetic acid (HAc) and 5 mM HAc in 90% acetonitrile. The gradient profile over time is shown in SI Table 2. The mobile phase for FdUMP and dTMP consisted of 0.1% HAc and 0.1% HAc in 100% acetonitrile (95:5). Elution was performed at a flow rate of 0.3 ml/min for 10 min. The injection volume was 40 µl, the temperature in the column oven was 30 °C, and the autosampler was kept at 8 °C.

### Preparation of total RNA and cDNA synthesis

Total RNA was isolated from 10  to  30 mg tissue using Qiagen AllPrep DNA/RNA/protein mini kit (no. 80004, Qiagen, Sollentuna, Sweden) according to the manufacturer’s instructions. The samples were kept at − 80 ℃ until analysis. cDNA was synthesized from total RNA using the High Capacity cDNA Reverse Transcription Kit (no. 4368814, ThermoFisher Scientific, Stockholm, Sweden) and run on a Bio-Rad thermal T100 cycler.

### Real-time quantitative PCR

Expression of the genes OAT2, TYMP, TK1, TYMS, ABCC5, and ABCC11, which are involved in 5-FU transport and metabolism, was quantified in tissue samples using real time qPCR. Gene assays are presented in SI Table 3. The qPCR was set up in 96-well plates as 10 µl reactions consisting of 5 µl 1 × TaqMan Gene Expression Mastermix (ThermoFisher Scientific, Stockholm, Sweden), 0.5 µl TaqMan gene expression assay (ThermoFisher Scientific, Stockholm, Sweden), 3.5 µl nuclease-free water, and 1 µl cDNA. The PCR was run on a 7500 fast real time PCR system (Applied Biosystems, Foster City, CA, USA). The PCR cycling parameters were 20 s at 95 °C, 40 cycles for 3 s at 95 °C, and 30 s at 60 °C. Each sample was run in duplicate and a mean Ct value was calculated. The Ct values of the target genes were related to the Ct values of the two endogenous house-keeping genes ACTB and GAPDH (SI Table 3).

### Statistics

The JMP 15.0.0/SAS software (SAS Institute Inc. Cary, NC, USA) was used for the statistical analyses. Data are presented as mean ± SD or median (range). Differences between groups were calculated using the Kruskal–Wallis’ test or the Pearson’s chi-squared test. To compare sets of continuous parameters measured in the same sample (matched pairs), the Pearson correlation coefficient (*r*) was calculated. A *p* value less than 0.05 was considered significant.

### Ethics

The study was conducted in accordance with the ethical standards established in the 1964 Declaration of Helsinki and its later amendments. The study was approved by the Regional Ethics Committee of University of Umeå, Sweden, Dnr 00-213, and all participants provided written informed consent before enrollment.

## Results

22 patients (12 men), median age 65 (49–75) years, BMI median 22.8 (19.3–30.7) with radiologically resectable PDAC in TNM stage IB (*n* = 13) and IIA (*n* = 9), were included during the study period 2005 to 2009. A laparoscopy was performed median 12 (3–35) days before pancreatic resection to implant an IP Port-a Cath. The night before scheduled pancreatic resection, an IP instillation of median 2225 (2050–2700) mg 5-FU was administered. The abdominal dwell time was acording to protocol median 640 (585–735) minutes in 19 patients, and median 205 (166–220) minutes in 3 patients due to late start of instillation. Surgery proceeded with a pancreatoduodenectomy (*n* = 13), total pancreatectomy (n = 3) or an exploratory laparotomy (*n* = 6). The tumours were high, intermediate or low differentiated pancreatic adenocarcinoma in 3, 16, and 3 cases, respectively. There were 13 R0 and 3 R1 resections. Six patients with liver metastases (*n* = 3) or non-resectable locally advanced disease (*n* = 3), had an exploratory laparotomy. Length of hospital stay (LOS) was median 16 (7–31) days. Overall survival was 335 (11–991) days with one death within 30 days of surgery. The patient characteristics and treatment details are presented in Table 1.

**Table 1:** Patient characteristics and treatment details

Case	Sex	Age (years)	BMI (kg/m^2^)	Stage (TNM)	ΔPAC (days)	5-FU (mg)	ΔIP (mins.)	ΔPlasma (mins.)	Surgery (procedure)	LOS (days)	Survival (days)
1	F	49	22.2	IB	2	2300	710	315	PD	13	407
2	M	50	25.3	IB	14	2550	690	337	PD	16	304
3	F	61	27.9	IIA	14	2150	630	296	PD	33	923
4	F	62	21.2	IIA	9	2100	617	139	EL	22	249
5	F	60	21.3	IB	3	2150	640	355	PD	26	752
6	M	62	21.0	IB	8	2400	627	164	EL	11	905
7	F	65	23.6	IIA	28	2100	585	NA	EL	16	366
8	F	64	24.6	IIA	14	2100	610	NA	EL	15	677
9	M	64	19.5	IIA	27	2700	655	NA	EL	14	134
10	M	65	22.7	IIA	16	2400	605	358	PD	15	301
11	M	67	24.3	IB	25	2400	645	303	PD	19	991
12	F	69	20.2	IIA	5	2050	696	430	PD	10	484
13	M	70	27.4	IIA	7	2500	590	295	PD	23	268
14	M	71	23.0	IIA	35	2600	632	372	PD	31	43
15	F	71	22.6	IB	14	2100	715	310	TP	22	198
16	F	70	19.3	IB	5	2100	205	NA	EL	14	31
17	M	72	22.6	IB	9	2300	640	360	PD	28	302
18	F	75	30.7	IB	25	2100	690	358	PD	17	657
19	M	75	22.9	IB	28	2150	666	290	PD	28	419
20	M	74	23.0	IB	7	2400	220	335	TP	12	11
21	M	61	22.2	IB	5	2150	166	304	PD	20	644
22	M	62	23.3	IB	5	2550	735	285	TP	29	152

*M*, male; *F*, female; *5-FU*, 5-flurorouracil given IP in mg; *ΔIP*, intraperitoneal dwell time of 5-FU; *ΔPAC*, duration in days from placement of intraperitoneal Port-a-cath to radical surgery; *TNM*, radiological preoperative stage according to AJCC Cancer Staging Manual 8th ed.; *ΔPlasma*, time in minutes between plasma sample at start of laparotomy and immediately after pancreatic resection or, if only exploratory laparotomy, when closing the abdomen; surgery, result of surgical procedure; *PD*, pancreatoduodenectomy; *TP*, total pancreatectomy; *EL*, exploratory laparotomy; *LOS*, length of hospital stay in days; Survival, survival after radical surgery in days; *NA*, not applicable

### Adverse events and postoperative complications

Adverse events of IP 5-FU that presumably could not be accounted to the surgical procedure during IP treatment, included grade 1 abdominal distension (91%), nausea (23%) and abdominal pain (10%). Two patients (case 7 and 15) with grade 2 abdominal pain during IP treatment was successfully treated with analgesics. No mucositis, diarrea, hand-foot syndrome or Port-a-cath infection was registered.

Two patients (case 3 and 17) experienced grade 2 anaemia, treated with two units of blood on POD2 and POD3. Two patients (case 18 and case 19) had a grade 2 cardiac complication, with a short hypotensive episode without chest pain or patterns of myocardial ischemia on ECG, on POD1 and POD10. One patient (case 5) had a grade 3A intraabdominal abscess caused by leakage from the pancreaticojejunal anastomosis, treated with percutaneous drainage on POD11. Three patients had a grade 3B gastrointestinal complication with hematemesis (case 20) or clinical deterioration (case 15 and 13) treated with relaparotomy on POD1, POD2, and POD12. Bleeding from the gastrointestinal anastomosis was revealed in one patient (case 20), and no obvious intraabdominal complication was found in the other two patients (case 15 and 13). One patient (case 20), developed sign of progressive cardiac failure on POD8, and died on POD11 in congestive cardiac failure, pulmonary edema, renal insufficiency and septicemia.

### LC–MS/MS analysis of plasma and tissue samples

5-FU, FdUr, and dUr were analyzed in plasma obtained before and after pancreatic resection. As shown in Table 2, the mean 5-FU level was decreased after resection (*p* < 0.0001), whereas the dUr level was increased (*p* = 0.014). However, no significant difference was seen when the FdUr levels were compared.

**Table 2:** Mean concentration of 5-FU and the metabolites dUr, FdUr, FdUMP, and dTMP in lymph node vs liver tissue before resection and pancreatic tumour vs pancreatic tissue after resection

Before resection	5-FU	FdUr	FdUMP	dUr	dTMP
Lymph node (pmol/g)	701 ± 678 (*n* = 14)	1.4 ± 1.5 (*n* = 14)	18.5 ± 15.5 (*n* = 13)	316 ± 273 (*n* = 14)	169 ± 150 (*n* = 13)
Liver tissue (pmol/g)	2229 ± 419 (*n* = 20)	1.1 ± 1.9 (*n* = 20)	6.1 ± 10.2 (*n* = 18)	52.3 ± 41.3 (*n* = 20)	81.8 ± 55.5 (*n* = 18)
*P*	0.27	0.15	0.017	0.0002	0.48
After resection	5-FU	FdUr	FdUMP	dUr	dTMP
Pancreatic tumour (pmol/g)	840 ± 2088 (*n* = 15)	1.4 ± 2.1 (*n* = 14)	6.7 ± 9.8 (*n* = 13)	132 ± 166 (*n* = 15)	105 ± 225 (*n* = 13)
Pancreatic tissue (pmol/g)	639 ± 1212 (*n* = 15)	0.79 ± 0.97 (*n* = 15)	1.6 ± 2.2 (*n* = 15)	210 ± 255 (*n* = 15)	25.1 ± 33.9 (*n* = 15)
*P*	0.84	0.67	0.068	0.30	0.11
Before and after resection	5-FU	FdUr	FdUMP	dUr	dTMP
Plasma before (pmol/ml)	904 ± 1362 (*n* = 19)	1.5 ± 5.2 (*n* = 19)	NA	122 ± 164 (*n* = 19)	NA
Plasma after (pmol/ml)	21 ± 60 (*n* = 18)	0.20 ± 0.32 (*n* = 18)	NA	269 ± 250 (*n* = 18)	NA
*P*	< 0.0001	0.71	NA	0.014	NA

Mean plasma concentration of 5-FU, dUr, and FdUr before vs after pancreatic resection

Lymph nodes and liver tissue from all patients were not sampled. Pancreatic tumour and pancreatic tissue was obtained if resection was performed. Plasma from 19 patients before and 18 patients after resection were sampled

*5-FU*, 5-flurorouracil; *dUr*, deoxyuridine; *FdUr*, 5-fluorodeoxyuridine; *FdUMP*, 5-fluorodeoxyuridine monophosphate; *dTMP*, deoxythymidine monophosphate; *NA*, not applicable; (*n*), patients

As expected, the 5-FU level in the wash fluid (*n* = 22) was lower than in the IP fluid (*n* = 22). The mean 5-FU concentration decreased from 729,093 ± 394,555 to 172,817 ± 344,041 pmol/ml. There was a strong, positive correlation between 5-FU levels in IP fluid and plasma obtained before resection (*r* = 0.82, *p* < 0.0001).

**Fig. 2:**
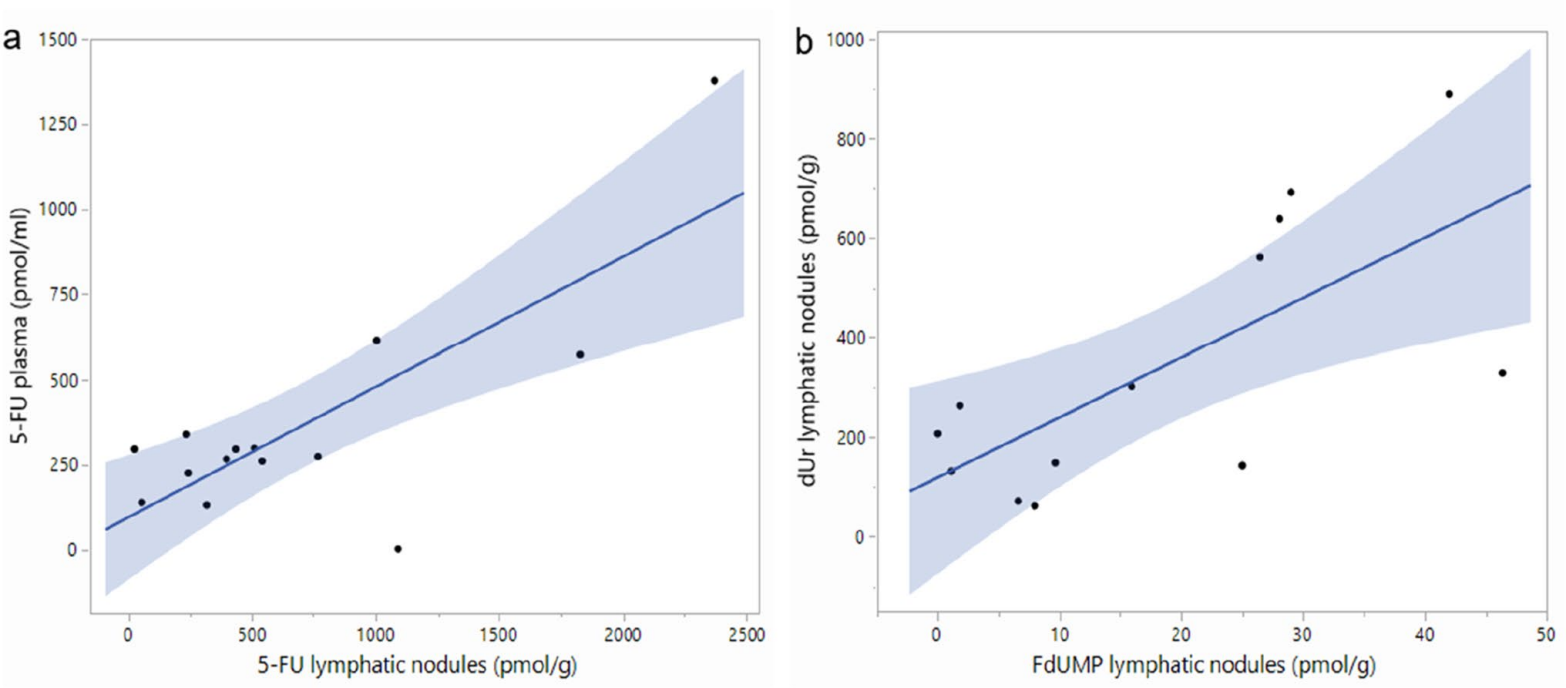
**a** Scatter plots showing a positive correlation between a) 5-FU levels in plasma and lymph nodes before resection (*r* = 0.78, *p* = 0.0009), and **b** between dUr and FdUMP levels in lymph nodes before resection (*r* = 0.70, *p* = 0.0076). The fit confidence region is shown as a blue-shaded area. *5-FU*, 5-flurorouracil; *dUr*, deoxyuridine; *FdUMP*, 5-fluorodeoxyuridine monophosphate

To determine if IP administration results in drug uptake in tissues with potential metastases from PDAC, the concentrations of 5-FU, dUr, FdUr, FdUMP, and dTMP were determined in lymph nodes and liver tissues sampled before pancreatic resection. As shown in Table 2, 5-FU was found in both lymph nodes and liver. There was a strong correlation between 5-FU plasma levels obtained before resection and levels in lymph nodes (*r* = 0.78, *p* = 0.0009, Fig. 2a). FdUMP reached the highest concentration in lymph nodes. There was a correlation between FdUMP and dUr levels in lymph nodes (*r* = 0.70, *p* = 0.0076, Figs. 2b and SI Fig. 1). The FdUMP level in liver correlated with plasma 5-FU (*r* = 0.59, *p* = 0.02) and, unexpectedly, there was a weak correlation between 5-FU and dTMP levels in liver samples (*r* = 0.49, *p* = 0.040).

The concentrations of 5-FU, dUr, FdUr, FdUMP, and dTMP were determined in pancreatic tumour and tissue sampled after resection (Table 2). IP administration resulted in high 5-FU concentrations in the tumours, although not as high as in the liver (*p* = 0.017). As shown in Table 2, the mean FdUMP level in tumours was higher (6.7 ± 9.8) compared to pancreatic tissue (1.6 ± 2.2), however, the difference did not reach significance (*p* = 0.068).

Plasma levels of dUr obtained before resection correlated positively with dUr in pancreatic tumours (*r* = 0.71, *p* = 0.006) as well as in pancreatic tissue (*r* = 0.84, *p* = 0.0006). The abdominal dwell time did not correlate with metabolite levels in plasma or tissue, and the levels of the three patients who had a short dwell time were within the same range as those of other patients.

### Gene expression analysis of tissue samples

**Fig. 3:**
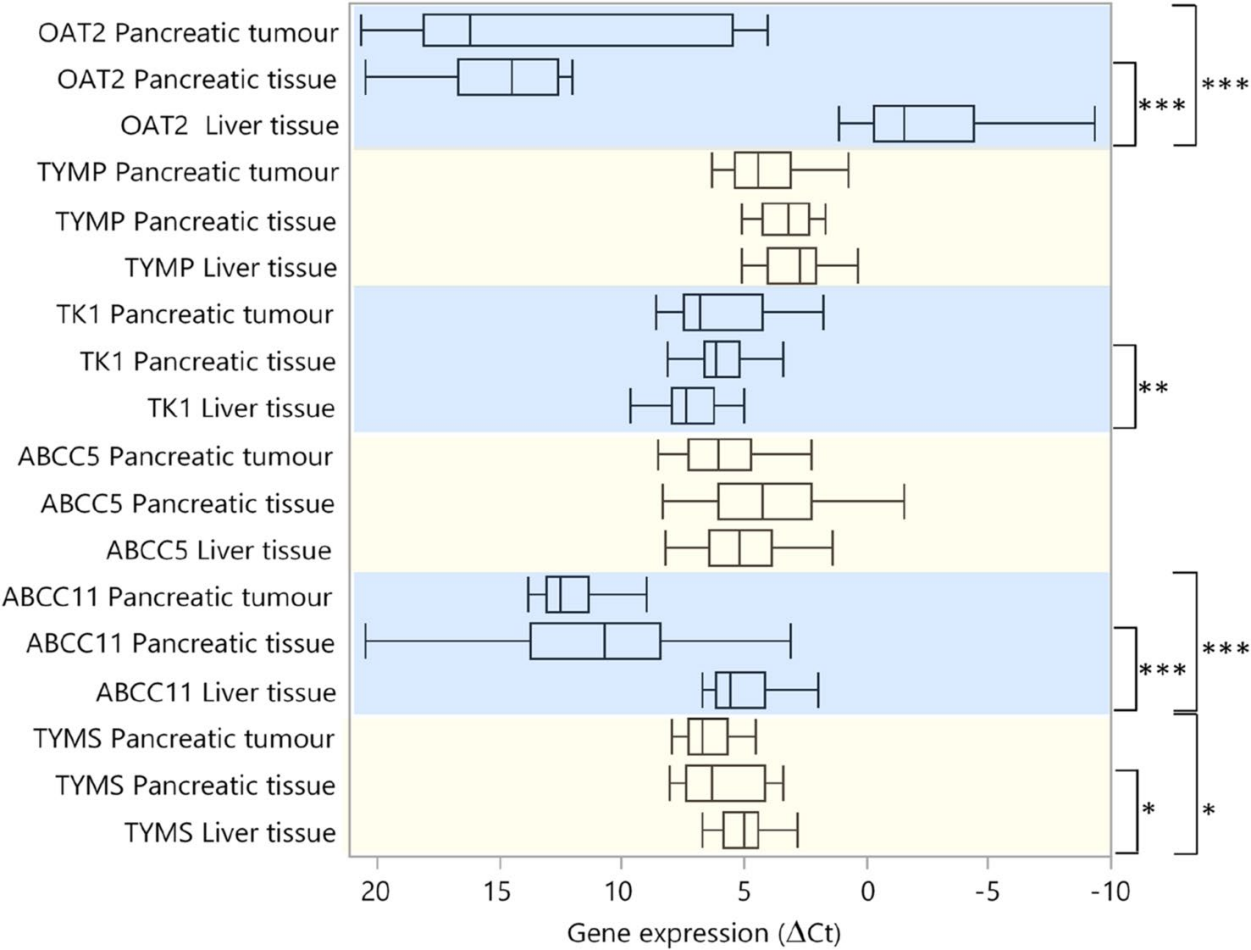
Comparison of gene expression in pancreatic tumour, pancreatic tissue, and liver tissue. The expression levels are presented as box plots with median values and ranges. Note that a high ΔCt value represents low gene expression and vice versa. The asterisks denote significance levels: *p ≤ 0.05, **p ≤ 0.01, ***p ≤ 0.00. *OAT2*, organic anion transporter 2; *TYMP*, thymidine phosphorylase; *TK1*, thymidine kinase 1; *ABCC5*, ATP-binding cassette subfamily C member 5; *ABCC11*, ATP-binding cassette subfamily C member 11; *TYMS*, thymidylate synthase

A panel of genes involved in transport and metabolism of 5-FU and its derivates was studied. The expression was analyzed in liver tissue, pancreatic tumour, and pancreatic tissue from 20, 14, and 16 patients, respectively. As shown in Fig. 3, the OAT2, ABCC11, and TYMS expression was significantly higher in liver compared to tumours, whereas the of ABCC5 and TK1 expression did not differ. In liver samples, OAT2 expression correlated positively with the expression of each analyzed gene, with the weakest correlation seen for TK1 (*r* = 0.54, *p* = 0.015) and the strongest for TYMS (*r* = 0.77, *p* < 0.0001). High TK1 expression correlated with high 5-FU levels in liver (*r* = 0.56, *p* = 0.013), and there was a positive correlation between TYMS expression and the dTMP level (*r* = 0.70, *p* = 0.0011).

**Fig. 4:**
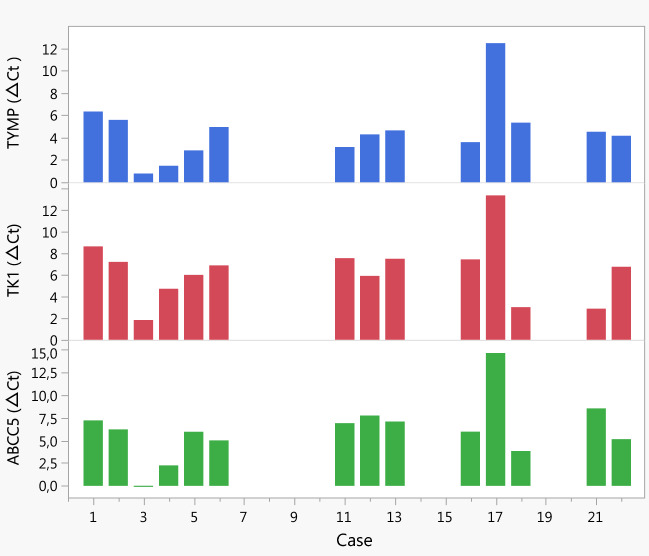
TYMP, TK1, and ABCC5 gene expression in pancreatic tumour of individual cases. Note that high ΔCt values correspond to low gene expression and vice versa. Pancreatic tumour tissue was not available for gene expression analysis from case 7–10, 14–15, and 19–20. *TYMP*, thymidine phosphorylase; *TK1*, thymidine kinase 1; *ABCC5*, ATP-binding cassette subfamily C member 5

The expression of the 5-FU influx transporter OAT2 was low in pancreatic tumour and pancreatic tissue compared to liver tissue (Fig. 3). The OAT2 expression varied greatly among tumour tissues, and when tumours were divided according to high/low OAT2 expression, those with high expression showed higher levels of FdUMP (*p* = 0.027). The tumours also had higher dTMP levels (*p* = 0.045) due to an exceptionally high level in one case. When excluding this case, no significant association between OAT2 expression and dTMP was seen whereas the positive association between OAT2 and FdUMP remained significant. There was a positive correlation between OAT2 expression in pancreatic tumour and 5-FU in plasma (*r* = 0.61, *p* = 0.036) as well as in IP fluid (*r* = 0.54, *p* = 0.049). Furthermore, a positive correlation was found between tumour expression of TYMP and TK1 (*r* = 0.77, *p* = 0.0013). Expression of these genes, which are involved in 5-FU conversion to FdUMP, also correlated with expression of the FdUMP efflux transporter ABCC5 (TK1 and ABCC5 *r* = 0.78, *p* = 0.001 and TYMP and ABCC5 *r* = 0.86, *p* < 0.0001) in pancreatic tumour. These correlations were reflected in individual samples (Fig. 4) but not in liver tissue, nor in pancreatic tissue (SI Figs. 2–4).

There was no significant difference in expression levels between pancreatic tumour and pancreatic tissue. However, TK1 expression was higher in pancreatic tissue compared to liver tissue (*p* = 0.0085). Only TYMS expression correlated between pancreatic tumour and tissue (*r* = 0.60, *p* = 0.032). No association between TYMS expression and FdUMP levels was seen in any tissue.

### Metabolite and gene expression levels by age, gender, and BMI

As mentioned, there were large individual differences in 5-FU concentrations in both plasma and IP fluid and it was noted that the highest levels were found in the two youngest patients. However, there was no general correlation between age and 5-FU, neither in plasma nor in IP fluid. The FdUr levels in liver were higher in males (*p* = 0.0047) whereas the dTMP levels in pancreatic tissues were higher in females (*p* = 0.032). Although the TYMS expression in pancreatic tissue was lower in females (*p* = 0.050), a positive correlation was seen between TYMS expression and dTMP levels in females (*r* = 0.79, *p* = 0.019). No association was found between the metabolite or gene expression levels and BMI.

## Discussion

As aggressive regimens for neoadjuvant chemotreatment and chemoradiotherapy are evaluated, it is important to identify a less toxic route for drug delivery. This study was performed to investigate uptake, distribution and metabolism of preoperative IP 5-FU and whether sufficient concentrations of 5-FU to inhibit TYMS, could be achieved in tissues along the metastatic route in lymph nodes and liver, as well as in pancreatic tumour and pancreatic tissue, in patients with PDAC. IP 5-FU could potentially be effective, yielding a higher total drug exposure (Area Under Curve) for the intraperitoneal compartments than attained if the drugs are given intravenously.

The results showed that after preoperative IP 5-FU, the drug and its metabolites could be detected in high concentrations in IP fluid and plasma, as well as along the metastatic route in lymph nodes, liver tissue, and furthermore in pancreatic tumour and pancreatic tissue. The concentration ranges of 5-FU and FdUMP in plasma and tissues (i.e. pmol/ml or pmol/g) were similar to those achieved in previous clinical pharmacokinetic studies [28, 29]. As expected, the 5-FU levels in plasma were significantly lower after tumour resection, as several hours had passed since the drug was evacuated. However, there was a large variation in plasma 5-FU levels among patients. This variation was not related to the BMI or age.

Pancreatic surgery as such is heavily burdened with postoperative complications [4]. In this study with preoperative IP 5-FU, complications according to Clavien–Dindo in grade 1 + 2 (18%) and 3 + 4 (14%) were comparable to average (34% and 14%), but in grade 5 (death of the patient), higher than average (4.5% vs 1.5%) [4]. The small number of patients increases the impact of one single event. Hypotensive episodes at POD1 after major intraabdominal surgery, due to low cardiac output or hypovolemia, is not uncommon, and is usually treated with aggressive intravenous fluid therapy and low dose intravenous norepinephrine.

All events after IP 5-FU instillation and before start of surgical procedure was allocated to the CTCAE adverse events. Different administration schedules of fluoropyrimidines can be of importance for the toxicity profile. In our experience [18], cardiac adverse events might occur in patients with no previous history of angina, first on the second day of two consecutive days of treatment with 1500 mg/m^2^ IP 5-FU, suggesting an accumulative effect in simulating a continuous infusion.

The levels of FdUMP and dUr in lymph nodes were significantly higher compared to the liver. In contrast, the 5-FU levels were non-significantly higher in liver compared to lymph nodes. This concurs with earlier findings of a considerable uptake of IP 5-FU in lymphatic vessels and lymph nodes draining the peritoneal mesothelium [16] and furthermore high levels of 5-FU in the portal and hepatic venous blood [30, 31] with rapid elimination by the first passage effect in the liver [32]. The correlation between FdUMP and FdUr in lymph nodes (Figs. 2b, SI Fig. 1), might indicate that IP 5-FU results in inhibition of the target enzyme TYMS.

The stromal component of the pancreatic tumour is higher than in many other GI-cancers [33], and often constitutes as much as 90% of the tumour mass [34]. The dense stromal matrix leads to vascular deficiency which might decrease tumoural concentration of 5-FU. Even so, after IP treatment, both 5-FU and FdUMP were detected in pancreatic tumour with a tendency towards higher concentration in pancreatic tumour compared to pancreatic tissue.

The large interindividual variation in 5-FU levels of and its metabolites may relate to differences in expression of genes involved in uptake, distribution, and metabolism of 5-FU. Aberrant expression of these genes, in addition to the dense stromal barrier, may lead to 5-FU drug resistance which is especially common in pancreatic cancer [35].

Previous studies have shown that the 5-FU influx transporter OAT2 is abundantly expressed in the liver, which is the central organ for drug metabolism and detoxification. In agreement with previous data [36], OAT2 was highly expressed in the liver whereas most of the pancreatic tumours and tissues had extremely low expression of the gene.

In accordance with OAT2 expression, the 5-FU levels were significantly higher in liver compared to pancreatic tumour and tissue. This indicates that tumours expressing OAT2 accumulate intracellular 5-FU and the active metabolite FdUMP.

The large variation in OAT2 expression among pancreatic tumours might be one reason for intracellular 5-FU differences. It is not known if some tumours had an intrinsically high expression of OAT2, or if transcription of the gene was induced in response to 5-FU. Other mechanisms, e.g., facilitated transport or non-facilitated diffusion of 5-FU may also contribute to the variation [37, 38]. Furthermore, rapid intracellular 5-FU degradation through the action of dihydropyrimidine dehydrogenase (DPD) may lead to low tissue levels of 5-FU [39] and development of drug resistance.

Inside the cells, 5-FU is converted to the active metabolite FdUMP by two main routes [21]. In the first step of the TYMP-TK1 route, TYMP catalyzes the conversion of 5-FU to FdUr (Fig. 1). Thus, high expression of TYMP is expected to result in high levels of FdUr. However, the FdUr levels were very low in plasma and tissue samples, making it hard to draw conclusions regarding FdUr in relation to the other metabolites. The low level may be caused by a rapid conversion of FdUr to FdUMP in the next step, catalyzed by TK1, or by generation of FdUMP through the OPRT-RNR route in which FdUr is not metabolized (Fig. 1).

High levels of FdUMP are needed to inhibit de novo dTMP synthesis through the action of TYMS. Although some pancreatic tumours with high FdUMP levels had low dTMP levels, others actually had high levels. This may be explained by the fact that when FdUMP binds the TYMS protein, autoregulation is inhibited, i.e., no downregulation of TYMS expression will occur through binding of the TYMS protein to its own mRNA [40]. Thus, in some tumours, 5-FU treatment may induce drug resistance by upregulating TYMS. Furthermore, high dUMP levels may prevent binding of FdUMP to the ternary complex, or shorten the duration of TYMS inhibition [41]. Another possible explanation could be activation of the salvage pathway, in which thymine is converted to thymidine by TYMP, followed by phosphorylation of thymidine to dTMP through the action of TK1 [21]. The positive correlation between TYMP and TK1 expression in pancreatic tumours supports this possibility. Interestingly, the expression pattern of ABCC5 in individual tumours closely followed that of TYMP and TK1. This means that tumours with the highest expression of TYMP and TK1, which would result in high FdUMP levels, also had high ABCC5 expression, which would result in efflux of FdUMP. A high efflux through upregulation of ABCC5 has been shown to occur in pancreatic cells and is a mechanism for 5-FU drug resistance [23, 24]. The ABCC11 gene, which was highly expressed in liver samples in addition to ABCC5, was expressed at a very low level in pancreatic tumour. Thus, it is not likely that FdUMP efflux through ABCC11 contributes to 5-FU drug resistance as much as ABCC5 in pancreatic cancer.

Since the salvage pathway utilizes free bases and nucleosides generated by degradation of DNA and RNA, it may be more active in cells with folate deficiency. Without adequate levels of folate, DNA synthesis, repair, and methylation are impaired. Folate deficiency is common in patients with pancreatic cancer where it leads to DNA hypomethylation [42] and upregulation of a number of genes [43]. Thus, one reason for TYMS overexpression in pancreatic tumour might be DNA hypomethylation [39, 44]. In addition, low levels of folate may affect generation of the ternary complex, where MeTHF is needed as a cofactor. Although intravenous folinic acid was given in combination with 5-FU, the dense stromal matrix might have prevented a rapid distribution to the tumour.

TYMS inhibition leads to a rise in the intracellular pool of dUMP and increased levels of dUr in tissues and blood [22]. The correlation found between dUr levels in plasma, pancreatic tumour, and pancreatic tissue as well as the increased dUr level in plasma after resection, indicates a possibility to use plasma dUr as a surrogate marker for TYMS inhibition. This might be useful to predict response during 5-FU treatment. Furthermore, since the liver is a common location for metastases from pancreatic cancer, it might be of value to investigate if plasma metabolites predict metabolite levels in liver. This study showed that 5-FU in plasma before resection correlated positively with the FdUMP level in liver samples.

### Strengths and limitations of the study

Recruitment of study participants was slow and there was a considerably delay between sampling and analysis. Due to the risk of complications, no tissue samples were taken during laparoscopy before start of treatment, thus no baseline values could be determined. Furthermore, in some patients where only exploratory laparotomy was performed, no pancreatic tumour or pancreatic tissue was obtained. Three patients were given the IP 5-FU early in the morning instead of late the night before surgery, resulting in shorter dwell time. However, the variation in time from infusion to sampling did not correlate with 5-FU in plasma or tissues and the metabolite and gene expression levels in the three patients was well within the spread of data. To ensure as optimal drug distribution as possible in the abdominal cavity, the 5-FU was dissolved in a volume of 2000 ml [45]. All tissues were rinsed in generous amounts of 0.9% NaCl to avoid biopsy contamination from IP 5-FU, although we cannot exclude minor influence on the 5-FU levels. Furthermore, variation in time from sampling of tissue to freezing might have affected the results. Genes of the OPRT-RNR routes for generation of FdUMP were not analyzed in the present study, which is a weakness, as is the lack of information regarding the DPD geno-phenotype. The activity of DPD may affect levels of 5-FU metabolites.

The strength of this study is that we show for the first time that IP 5-FU in resectable PDAC results in drug uptake and metabolism in tumour tissue and in the most common locations for metastases, the liver and lymph nodes.

## Conclusion

This study shows that IP 5-FU can penetrate pancreatic tumour and adjacent tissues along the metastatic route, and metabolize to FdUMP which is needed for inhibition of TYMS. There was a correlation between the FdUMP and dUr levels in lymph nodes indicating that IP 5-FU results in inhibition of the target enzyme TYMS. The large individual differences in levels of 5-FU and its metabolites may be related to expression of genes involved in transport and metabolism. Tumours with high expression of the influx transporter OAT2 may accumulate the metabolite FdUMP. Extended studies are warranted to evaluate the IP route for 5-FU administration in PDAC patients.

## Consent for publication

Permission has been obtained from all named authors to submit the manuscript for publication.

## Supplementary Information

Below is the link to the electronic supplementary material.

Supplementary file1 (DOCX 15 kb)

Supplementary file2 (DOCX 150 kb)

## Data Availability

The data generated or analyzed during the current study are included in this published article and its supplementary information files.

## Abbreviations

5-FU: 5-Fluorouracil
ABCC5/MRP5: ATP-binding cassette, subfamily C (CFTR/MRP), member 5
ABCC11/MRP8: ATP-binding cassette, subfamily C (CFTR/MRP), member 8
ACTB: β-Actin
AJCC: American Joint Committee on Cancer
ATP: Adenosine triphosphate
BMI: Body mass index
CFTR: Cystic fibrosis transmembrane conductance regulator
CldUr: Chlorodeoxyuridine
Ct: Cycle threshold
CTCAE: Common terminology criteria for adverse events
DNA: Deoxyribonucleic acid
dTMP: Deoxythymidine monophosphate
dUMP: Deoxyuridine monophosphate
dUr: Deoxyuridine
EDTA: Ethylenediaminetetraacetic acid
FdUDP: Fluorodeoxyuridine diphosphate
FdUMP: 5-Fluorodeoxyuridine monophosphate
FdUr: 5-Fluorodeoxyuridine
FLV: 5-Fluorouracil + leucovorin
FLOX: FLV + oxaliplatin
FOLFIRINOX: Folinic acid, fluorouracil, irinotecan, oxaliplatin
FUDP: Fluorouridine diphosphate
FUMP: Fluorouridine monophosphate
FUTP: Fluorouridine triphosphate
GAPDH: Glyceraldehyde-3-phosphate dehydrogenase
HAc: Acetic acid
IP: Intraperitoneal
ISGPS: International study group for pancreatic surgery
LC–MS/MS: Liquid chromatography with tandem mass spectrometry
LOS: Length of hospital stay
MeTHF: Methylenetetrahydrofolate
mRNA: Messenger ribonucleic acid
MRP: Multidrug resistance protein
NaCl: Sodium chloride
OAT2/SLC22A7: Organic anion transporter 2/solute carrier family 22, member 7
OPRT: Orotate phosphoribosyl transferase
PAC: Port-a-cath
PDAC: Pancreatic ductal adenocarcinoma
POD: Postoperative day
qPCR: Quantitative polymerase chain reaction
RNA: Ribonucleic acid
RNR: Ribonucleotide reductase
TK1: Thymidine kinase 1
TNM: Tumour, node, metastasis
TYMP: Thymidine phosphorylase
TYMS: Thymidylate synthase

## Code availability

JMP 15.0.0/SAS software (SAS Institute Inc. Cary, NC, USA) was used for the statistical analyses.
