# Supplementary material for: Pharmacokinetics of preoperative intraperitoneal 5-FU in patients with pancreatic ductal adenocarcinoma
Source: Cancer Chemother Pharmacol. 2021 Jun 16;88(4):619–31. doi: 10.1007/s00280-021-04318-x (PMC8367903; doi:10.1007/s00280-021-04318-x)
Supplement: Supplementary file 1 — Supplementary file1 (DOCX 15 kb) [file 280_2021_4318_MOESM1_ESM.docx]

**SUPPLEMENTARY FIGURES**

**SI Fig. 1** Levels of metabolites in lymph nodes of individual cases. Tissue from lymph nodes was not available for analysis from case 1-3, 11, 14-15, and 17-18. 5-FU, 5-flurorouracil; dUr, deoxyuridine; FdUr, 5-fluorodeoxyuridine; FdUMP, 5-fluorodeoxyuridine monophosphate; dTMP, deoxythymidine monophosphate

**SI Fig. 2** Metabolite and gene expression levels in liver tissue of individual cases. Note that high ΔCt values correspond to low gene expression and vice versa. OAT2 expression was high in all samples and the expression of ABCC11 was higher than the expression of ABCC5, with the exception of case 13. Liver tissue was not available for metabolite analysis from case 1-2, and for gene expression analysis from case 1 and 5. 5-FU, 5-flurorouracil; dUr, deoxyuridine; FdUr, 5-fluorodeoxyuridine; FdUMP, 5-fluorodeoxyuridine monophosphate; dTMP, deoxythymidine monophosphate OAT2, Organic anion transporter 2; TYMP, Thymidine phosphorylase; TK1, Thymidine kinase 1; ABCC5, ATP-binding cassette subfamily C member 5; ABCC11, ATP-binding cassette subfamily C member 11; TYMS, Thymidylate synthase

**SI Fig. 3ab** Metabolite and gene expression in pancreatic tumour of individual cases. Note that high ΔCt values correspond to low gene expression and vice versa. a) The metabolite and gene expression levels varied greatly with the exception of ABCC11 expression which was consistently low. The tumour of case 3 had comparatively high levels of 5-FU and dTMP, and also the highest expression of TYMP, TK1, and ABCC5. b) Variation in 5-FU and dTMP levels after exclusion of case 3. Pancreatic tumour tissue was not available for metabolite analysis from case 2, 9-10, 14-15, and 19-20, and for gene expression analysis from case 7-10, 14-15, and 19-20. 5-FU, 5-flurorouracil; dUr, deoxyuridine; FdUr, 5-fluorodeoxyuridine; FdUMP, 5-fluorodeoxyuridine monophosphate; dTMP, deoxythymidine monophosphate; OAT2, Organic anion transporter 2; TYMP, Thymidine phosphorylase; TK1, Thymidine kinase 1; ABCC5, ATP-binding cassette subfamily C member 5; ABCC11, ATP-binding cassette subfamily C member 11; TYMS, Thymidylate synthase.


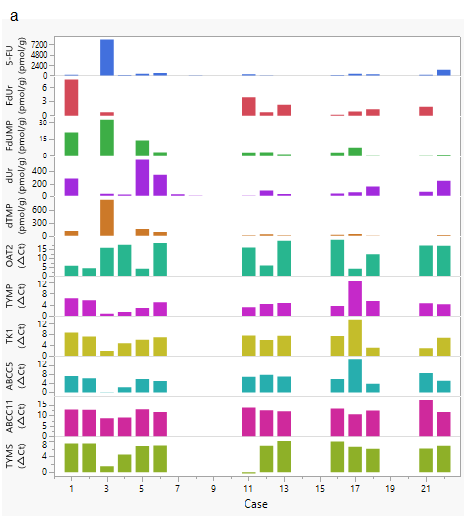

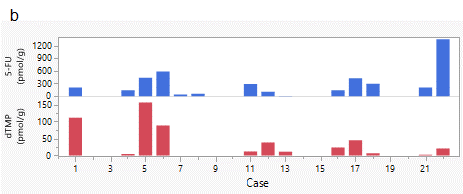


**SI Fig. 4** Metabolite and gene expression levels in pancreatic tissue of individual cases. Note that high ΔCt values corresponds to low gene expression and vice versa. In contrast to pancreatic tumour, there was a large variation in ABCC11 expression in pancreatic tissue. Pancreatic tissue was not available for metabolite analysis from case 1-2, 9-10, 15, and 19-20, and for gene expression analysis from case 2, 9-10, 15, and 19-20. 5-FU, 5-flurorouracil; dUr, deoxyuridine; FdUr, 5-fluorodeoxyuridine; FdUMP, 5-fluorodeoxyuridine monophosphate; dTMP, deoxythymidine monophosphate OAT2, Organic anion transporter 2; TYMP, Thymidine phosphorylase; TK1, Thymidine kinase 1; *ABCC5*, ATP-binding cassette subfamily C member 5; ABCC11, ATP-binding cassette subfamily C member 11; TYMS, Thymidylate synthase
