# Supplementary material for: Pharmacokinetics of preoperative intraperitoneal 5-FU in patients with pancreatic ductal adenocarcinoma
Source: Cancer Chemother Pharmacol. 2021 Jun 16;88(4):619–31. doi: 10.1007/s00280-021-04318-x (PMC8367903; doi:10.1007/s00280-021-04318-x)
Supplement: Supplementary file 2 — Supplementary file2 (DOCX 150 kb) [file 280_2021_4318_MOESM2_ESM.docx]

**SUPPLEMENTARY TABLES**

**SI** **Table 1** List of compounds and MS/MS acquisition parameters

|  | Precursor ion (m/z) | Product ion  (m/z) | Cone voltage (V) | Collision energy (V) |
| --- | --- | --- | --- | --- |
|  |  |  |  |  |
| 5-FU | 128.9 | 41.7 | 27 | 13 |
| FdUr | 245.0 | 155.1 | 27 | 15 |
| FdUMP | 325.0 | 195.0 | 25 | 13 |
| dUr | 227.1 | 184.1 | 27 | 11 |
| CldUr | 261.0 | 171.0 | 29 | 15 |
| dTMP | 321.0 | 195.0 | 25 | 20 |

MS/MS, tandem mass spectrometry; m/z, mass-to-charge ratio; V, volt; 5-FU, 5-flurorouracil; FdUr, 5-fluorodeoxyuridine; FdUMP, 5-fluorodeoxyuridine monophosphate; dUr, deoxyuridine; CldUr, chlorodeoxyuridine; dTMP, deoxythymidine monophosphate

**SI Table 2** Gradient elution profile flow^a^

| Time | Mobile phase | |
| --- | --- | --- |
| (minutes) | A (%) | B (%) |
| 0 | 100 | 0 |
| 1 | 95 | 5 |
| 2 | 92 | 8 |
| 3 | 30 | 70 |
| 4 | 30 | 70 |
| 4.4 | 10 | 90 |
| 5 | 0 | 100 |
| 6 | 0 | 100 |
| 6.5 | 100 | 0 |
| 10 | 100 | 0 |
| ^a^ 0.25 ml/minute | | |

**SI Table 3** List of analyzed genes

| **Gene category** | **Gene** | **Gene name** | **Assay ID** |
| --- | --- | --- | --- |
|  |  |  |  |
| **5-FU influx** | *OAT2/SLC22A7* | Organic anion transporter 2/Solute carrier family 22, member 7 | Hs00198527_m1 |
| **5-FU metabolism** | *TYMP* | Thymidine phosphorylase | Hs00157317_m1 |
|  | *TK1* | Thymidine kinase 1 | Hs01062125_m1 |
|  | *TYMS* | Thymidylate synthase | Hs00426586_m1 |
| **FdUMP efflux** | *ABCC5/MRP5* | ATP-binding cassette, subfamily C (CFTR/MRP), member 5 | Hs00981089_m1 |
|  | *ABCC11/MRP8* | ATP-binding cassette, subfamily C (CFTR/MRP), member 11 | Hs01090758_m1 |
| **House-keeping** | *ACTB* | β-actin | Hs99999903_m1 |
|  | *GAPDH* | Glyceraldehyde-3-phosphate dehydrogenase | Hs99999905_m1 |

5-FU, 5-flurorouracil; FdUMP, 5-fluorodeoxyuridine monophosphate; ATP, adenosine triphosphate; CFTR, cystic fibrosis transmembrane conductance regulator; MRP, multidrug resistance protein
